# Typhus Disease in Iran during the Qajar Period (1725 to 1925 AD); a Brief Historical Review

**DOI:** 10.34172/aim.2022.120

**Published:** 2022-11-01

**Authors:** Seyyed Alireza Golshani, Ghobad Mansourbakht, Faranak Alembizar

**Affiliations:** ^1^Postdoctoral Researcher, History of Iran after Islam, Department of History, Shahid Beheshti University, Tehran, Iran; ^2^Associate Professor, Department of History, Shahid Beheshti University, Tehran, Iran; ^3^Department of History of Medicine, School of Persian Medicine, Tehran University of Medical Sciences, Tehran, Iran

**Keywords:** Iran, Medical History, Typhus, World War I

## Abstract

Typhus is an acute febrile disease caused by a series of bacteria called Rickettsia that is transmitted by insects such as lice, fleas, and ticks. This disease has appeared several times in Iran and caused many casualties. There were some therapeutic measures taken by European physicians in Tehran and medical graduates of the Dar al-Fonun school or expatriates who had studied medical courses in Western countries, even though the taken steps were not enough. Due to the lack of sanitation and cleaning products after the outbreak of World War I in March 1917 and its synchronization with the swift outbreak of Typhus in 1918, heavy casualties followed. In this study, we first examine the prevalence of Typhus in the Qajar dynasty in Iran, and will then focus on the pathological importance of this disease history in Iran. After that, we will study the role of Typhus prevalence and World War I in the Persian famine, malnutrition, and food poverty. Moreover, we investigated the role that this great war had in strengthening the spread of this disease and its role in the death of many Iranian people.

## Introduction

 Infectious diseases have been the two major causes of abnormal human death throughout history. The Spanish flu was one of the most contagious and epidemic diseases which posed a great danger across Europe and human societies. It should be noted that this disease provided the ground for the emergence of another disease called typhus, which entered Iran and affected its people.^1^

 Even though the main epidemic of this disease began in 1917, in Iran’s modern history, only its peak between 1939 and 1941 has been considered. Unfortunately, the synchronicity of famine, cholera, Spanish flu, and typhoid fever with this disease has marginalized the role of typhus in other factors, especially the Spanish flu caused less attention in this regard. The new generation of typhus appeared in Poland and Germany during World War I and then entered Iran via Russia and the Ottoman Empire.^1,2^

 However, typhus has existed as a kind of endemic disease in Iran and the society had to deal with it seasonally, mainly based on old medical knowledge because of its emergence and peak in March and April and sometimes in autumn. Although the death caused by this type of typhus was limited, the spread of a novel generation of typhus in Iran during World War I exposed the country to a new type of typhus whose epidemic power was not comparable to the endemic typhus. For this reason, the number of affected typhus cases and its death rate in Iran increased significantly.^2^

 Typhus is an acute and febrile disease that arises from a bacterium called Rickettsia and is transmitted to humans by insects such as lice, fleas, and ticks. This disease could not be spread from person to person but is transmitted to humans through infected clothes, quilts, mattresses, carpets, mice, and insects. Typhus may affect the skin, central nervous system, gastrointestinal tract, and muscles.^3^ Generally, three types of typhus could be induced by fleas, ticks, and lice:

- Epidemic typhus, also called louse-borne typhus which is caused by rickettsia and is known to be the most common type of typhus.
- Endemic typhus or murine typhus which is caused by *Rickettsia typhi.*
- The third type is tick typhus caused by *R. australis.*^4^

When we talk about the epidemic of typhus during the Qajar dynasty during World War I, we often mean louse-borne typhus. The incubation period for typhus is between 1 and 2 weeks which usually takes about 12 days. Rickettsiae are small bacteria that are obligate parasites inside the cell. The carrier insects are lice in epidemic typhus, fleas in endemic typhus, and mites in Queensland tick typhus. The most common symptoms are fever, chills, muscle aches, headaches, severe weight loss, and skin rashes (with or without scars). In all three types of typhus, antibiotics such as tetracycline, doxycycline, or chloramphenicol are used as treatment. Observing a personal hygiene routine is a crucial measure in controlling epidemics.^5^

 The present study’s background on typhus in the Qajar dynasty in Iran is not as deep as it should be. The book by Willem Floor; “*Public health in Qajar Iran*”, and also, the paper titled “An Overview of Epidemic Typhus in the World and Iran during the 19th and 20th Centuries” by Azizi et al in the *Archives of Iranian Medicine* in October 2016, did not pay much attention to the issue of typhus during the Qajar dynasty in Iran.^6,7^

 The question that arises from this research and the researchers want to answer is that although endemic typhus in the Qajar dynasty was the most prevalent disease, it has not been addressed in numerous studies. In this study, we tried to find the causes and factors of the endemic typhus in Iran in a descriptive historiographical way, and then evaluate the dimensions of the effects of the typhus epidemic in the First World War in Iran.

## Typhus Epidemic or Indigenous Typhus in the Qajar dynasty in Iran

 Endemic typhus or indigenous typhus existed in Iran during the Qajar dynasty and appeared in winters and early springs. According to Dr. Willem Floor, typhus in Iran, although rare, was considered an endemic disease on a national scale. Typhus skin rash (with or without scarring) occurred throughout the country; the disease often took an endemic form in autumn and winter (especially in children); it was uncommon in young people but recurred in the elderly, which was considered the second wave of the disease. Contaminated drinking water was the main cause.^6^

 According to Dr. Jakob Eduard Polak (1818–1891), “typhus often appears as an epidemic in the winter months. Depending on whether the disease is mild or malignant, it is called typhus or typhoid fever. In Iran, this disease never appears ventricular, so the abdomen is only slightly swollen and there is no pain when pressing on the area of the appendix, on the contrary, constipation is always a complication of the disease. Typhus is highly contagious; in 1854, when I hospitalized several typhus patients in a hospital ward under my supervision where there were other patients, such as eye patients and the wounded, not only did all the patients develop typhus, but I and twelve of my sixteen students also got typhus and the remaining four students had been infected before”.^8^ Dr. Polak also says, “Typhus is more dangerous and appears with symptoms of blood infection and blood flows often from the nose and even viscera so that the teeth, lips, and tongue are covered with blood clots. The pulse is very slow and unsteady, the patient is under general anesthesia. The crisis begins 10 days after the disease, which is often associated with local sweating on the chest, without pain relief. After continuous bowel incontinence, death often occurs before the fourteenth day and sometimes on the eleventh day”.^8^

 Heinrich Karl Brugsch (1827–1894), the German orientalist, describes the areas around Anzali in northern Iran as follows, “The presence of various types of vermin is another feature of the southern shore of the Caspian Sea, and if we add the prevalence of indigenous fever, there is almost nothing left to say”.^9^ In addition to Anzali, Polack also considers other parts of the Caspian coast, e.g., Rasht and Talesh, as dangerous areas that cause typhus, fever, and eventually death in non-indigenous people passing through these areas. When the Larijan military force consisting of residents from Damavand, a mountainous region located four miles from Tehran, was transferred to Tehran, only less than a tenth of them survived due to fever and typhus. Of the large number of Gilani pilgrims who go to the Tomb of Shiite Imams in Mesopotamia every year, about one-third die during the journey.^8^ In addition to the north of the country, this disease was also present in other parts of Iran, e.g., Shiraz, which had an unhealthy environment despite its mild weather and unique verdancy, and various diseases and typhus were prevalent there.^9^ Typhus and fever-prone area of Fars starts from the place of growth of date palm tree and continues to the shores of the Persian Gulf. In the southern areas, it continues from Bushehr port to Bandar Abbas. In the Larestan county, the conditions are much more dangerous and there are large mosquitoes that cause a variety of febrile diseases.^9,10^

 In the province of Azerbaijan, typhus had locally been present, which is a type of contagious fever transmitted by lice. Another type of fever is transmitted by the Mianeh lice or “Gharib Gaz”.^11^ Lady Mary Leonora Woulfe Sheil writes during her trip through Tabriz to Mianeh, “after two days, we reached Mianeh. The city is famous for its ticks, and since one bite is enough to infect and gradually kill a person, we spent half the night taking precautionary measures to protect ourselves against the fever, which is one of its side effects and is prevalent across this insanitary area”.^12^ Ernest Orsolle, the Belgian tourist, reported on typhus and typhoid fever of Mianeh, “in Mianeh, there is an insect called “Mele of Mianeh”. This tiny insect is commonly known as *Argas persicus* but is more dangerous than its European types. The bite place is very painful and swollen, which sometimes causes typhus or typhoid fever and even death”.^13^

 Conte de Sercey, Ambassador Extraordinary and Plenipotentiary of France in 1840, stated on the global reputation of the “Mele of Mianeh” that, “Mianeh” is famous among travelers because of a type of insect called “Mele”. This insect is deadly and is said to be present in large numbers in the home. All the books and travelogues written in English about traveling to Iran have mentioned these insects with horror. But I believe this horror is a bit exaggerated...”.^14^

 Rezā Qoli Khān Hedāyat (1800–1871) was bitten by “Mele of Mianeh” in 1862. He fully describes the treatment of Iranian physicians toward his condition, saying that he was anesthetized several times, his pulse and heart rate were very slow due to the poison and he was about to die, but was finally treated by Hakim-Bashi (Physician) Fakhr al-Atebba.^14^

 Actually, “*Argas persicus*” is a small soft-bodied tick that is found primarily on domestic fowl such as chickens, ducks, and geese. It was first recorded by Lorenz Oken (1779–1851) German biologist and ornithologist in 1818 in Mianeh, Persia, and named *Rhynochoprion persicum.*^11,15^

 Dr. Jakob Eduard Polak, the Austrian medical teacher at the Dar Al-Fonun School, mentions the typhus epidemic in 1859.^16^ According to Joseph Désiré Tholozan, special physician of Naser al-Din Shah Qajar (Reign: 1848–1896), a severe outbreak of typhus reappeared in Tehran between 1864 and 1865.^6^ Moreover, at the end of winter and the beginning of spring of 1857 and 1874 during the reign of Nasser al-Din Shah Qajar, the outbreak of typhus among the barracks of Tehran caused casualties in this less equipped army due to the lack of basic health requirements.^17^ One of Dr. Polack’s students and medical graduates of Dar al-Fonun School tried to treat and control the disease by quarantining the army hospital.^17^

 In his book “*The Story of My Life*”, Abdollah Mostofi (1876–1950), the literate, writer and politician of the Qajar and Pahlavi eras, provides some information about a typhus epidemic in Tehran in the spring of 1872, “In the winter, there was a huge snow and rain, which closed the roads in some places caused more deaths. In the spring, typhoid and typhus killed many people, both rich and poor. This was the first time that Naser al-Din Shah faced such an event during his reign”.^18^ Once again, Mostofi reports on the outbreak of the disease in Tehran in the spring of 1880, which coincided with the famine, “During this famine, no one died of starvation, but the deaths were mostly from typhus and typhoid fever so that it often happened that all the people of a house were infected with this disease. Although there were no statistics recorded on the number of deaths in Tehran, it certainly reached 100–200 deaths per day, and this situation began in mid-winter and lasted until late spring and early summer”.^18^

 The disease continued in June 1898, during the reign of Mozaffar ad-Din Shah Qajar (1896–1907), as one of the Shiite clerics and imitation references, Mirza Ahmad Mojtahed Naraghi Kashani, who lived and taught in Atbat-e-Aliat, was infected by the disease and passed away during his trip to Mashhad upon entering the city of Semnan.^19^ Another important event during the reign of Mozaffar ad-Din Shah was the outbreak of typhus in Sistan. In a book entitled “Afghanistan” by Hamilton Angus (1874–1913), the author refers to the mission of Sir Vincent Arthur Henry McMahon in Sistan and Baluchestan of Iran that was under United Kingdom rule at the time. He reported, “In the early summer of 1904, typhus was found in Sistan,” the United Kingdom official wrote of typhus. “For a few months, this illness made a fuss! How many people died, no one will ever be able to understand, but the casualties were certainly very high”.^20^ Also, in the province of Azerbaijan, the typhus outbreak in Urmia caused the death of Dr. Joseph Plumb Cochran, (1855-August 18, 1905) physician, hospital manager, medical school and American missionary at the age of 50.^21^

 During the reign of Ahmad Shah Qajar (1909-1925) in Iran, tragic events such as World War I and numerous outbreaks of typhus from Russia to Iran occurred, which we will mention in chronological order. In November 1909, Russian typhus entered Bandar-e Gaz (Golestan Province in northern Iran), leading to casualties in the region.^7^

 The author of the book “The Story of Tehran” states about the typhus outbreak during the famine period in April 1910 and its consequences in Tehran, “In the winter, there was countless snow and rain, it blockades the roads in some areas and caused many deaths. In the spring, many people, both rich and poor, were died because of typhoid and typhus”.^22^

 In the same year, it seems that there was also an epidemic in Azerbaijan in northwestern Iran, as Ahmad Kasravi (1890-1946), who witnessed the event, states in his book “18-year history of Azerbaijan”, “In the winter of 1910, in addition to the famine, typhus also spread in Tabriz, Urmia, and Salmas, causing the death of many people”.^23^

## Typhus Epidemic in World War I in Iran

 World War I began in June 1914 on the pretext of the assassination of Archduke Francis Ferdinand, Crown Prince of Austria-Hungary. This time coincided with eight days after Ahmad Shah’s coronation when the Iranian government declared Iran to be neutral in the war.^24^ However, this neutrality was violated due to the weakness of the new Qajar king. Russian forces were present in the north, United Kingdom forces in the south and southeast, Ottoman forces in the west, and German forces in central and other areas of Iran.^24,25^ While the world was burning in the flames of World War I, a typhus epidemic appeared. Between 1918 and 1922, typhus caused at least 3 million deaths out of 20–30 million cases. In Russia after World War I, typhus killed three million people, largely civilians.^7,26^ The outbreak of the typhus epidemic was predictable during World War I in March 1917 due to the lack of detergents and hygiene products in Iran. This epidemic probably reached Iran from Tsarist Russia in the northern neighborhood of Iran by Polish immigrants and World War I Allied forces.^7,27^

 Dr. Mohammad Gholi Majd, the Iranian historian, takes a critical look at the loss of eight million people in Iran during World War I and highlights United Kingdom’s significant role in the prevalence of the disease and Persian famine of 1917–1919. He submitted his manuscripts based on documents available at the US State Department.^28^ According to Dr. Majd, “The onset of World War I and the presence of foreign forces caused political and economic instability, famine, poverty, and disease in Iran. This situation even worsened in the fall of 1917 and the winter and spring of 1918. This time, typhus and typhoid were accompanied by widespread famine, adding to the misery of the Iranian people, so that the people had to eat grass, dogs, and carcasses of animals and even humans. The Persian famine of 1917–1919 was not specific to small or large cities, spreading throughout the country from Tehran to Shiraz and from Tabriz to Mashhad. The normal death number in Tehran was between ten and fifteen people a day, which after the occurrence of this situation, this number increased to about one hundred and eighty people, indicating the deteriorating situation in Iran at those times. Things were getting to the point where people were buried in mass graves. Wherever the famine ended, typhus and cholera took their place. The reduction of Tehran’s population from 359 000–499 000 in 1899 to 299 000 in 1921 will leave no room for ambiguity.^6,29^ According to historical reports, the population of Iran had considerably reduced to about 6 million due to famine and deadly diseases such as typhus, cholera, and plague. In 1888, General Schindler estimated the country’s population at 7 653 600”.^30^

 Ein al-Saltanah, Qahraman Mirza Salur (1872–1945), was the nephew of Nasser al-Din Shah Qajar (1831–1896). In his book, he has provided a detailed description of the typhus epidemic in Iran. For example, he states about the events on March 19, 1919, “spring was a sign of hope and aspiration, but only for those who could save their lives and their families until the end of the harvest. Various diseases spread, and typhus, typhoid, and malaria were highly prevalent. There was horrible news from far and near provinces, and one misfortune came after the other...”.^31^

 He also added, “Various diseases such as cholera, typhus, and typhoid, etc. have invaded Iran, killing the poor people like a sickle harvesting the product. People are extremely anxious, fearing that cholera will reach Tehran. It has finally reached Qom. He writes in the newspaper that, in the winter and spring of that year, people spent lots of money on medicine and treatment, so some families even handed over everything they had from money, carpets, copper, etc. to physicians and pharmacies. The government, in turn, has spent a lot of money, but the disease has not yet been eliminated”.^31^ Because of his presence in Qazvin, Ein Al-Saltanah has pointed to the outbreak of the disease in the Alamut district of Qazvin, “The deputy governor of Alamut had written a description of typhoid fever and typhus in Alamut. He reported the death of 100 people and the serious illness of 150 people after 15 days. He had asked the government to provide more medicines and physicians. This disease is also rising in Rudbar; where is the medicine, where is the physician?”.^31^

 After the First World War I in the late period of the Qajar dynasty, several typhus outbreaks were reported in northeastern Iran in Mashhad and northwestern Iran in Hamedan, Azerbaijan, and Urmia, which had borders with Russia. Around 1920, it was reported that various infectious diseases, including typhus, were prevalent in rural areas of Hamedan. At those times, typhus was more common in autumn and winter, and children were at higher risk for the disease.^7^ Another case is the outbreak of typhoid and typhus in Ashgabat, Turkestan, Russia, which is reported in March 1921 documents. To prevent its spread to Iran, therefore, the parliament sent instructions to the Khorasan Provincial Commission to “publish self-protection instructions among the residents to prevent the spread of this disease”.^32^ In 1922, typhus broke out in the Azerbaijan province in northwestern Iran and killed 163 people between the years 1922 and 1923.^7^ Another case is the prevalence of typhus in Urmia and Tabriz on July 31, 1924. According to the report of the governor of Azerbaijan, typhus has become common in Urmia and some cases have also been observed in Tabriz. In Tabriz, the parliament gathered the physicians of the city and took the necessary measures to prevent the spread of typhus. The government of Urmia was also recommended to take similar measures.^25^ Eventually, the disease reached Tehran via the northeast and northwest, killing about 121 people between 1923 and 1924.^7^

**Figure 1:**
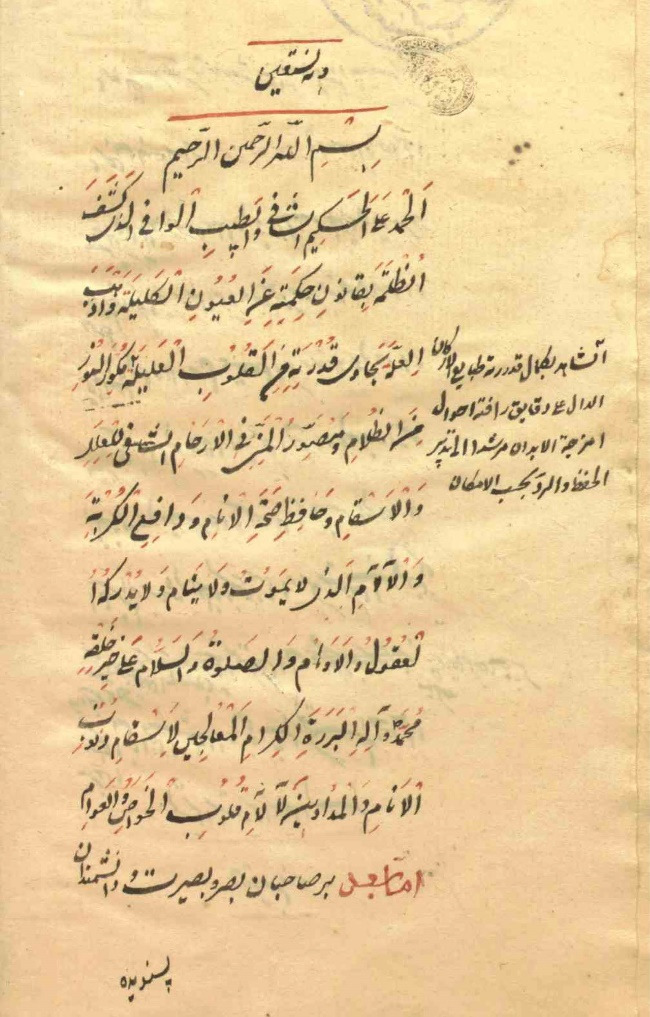
Tohfe-ye Naseri (1862) by Fakhr al-Atebba. In this book, he addressed typhus.^33^

**Figure 2:**
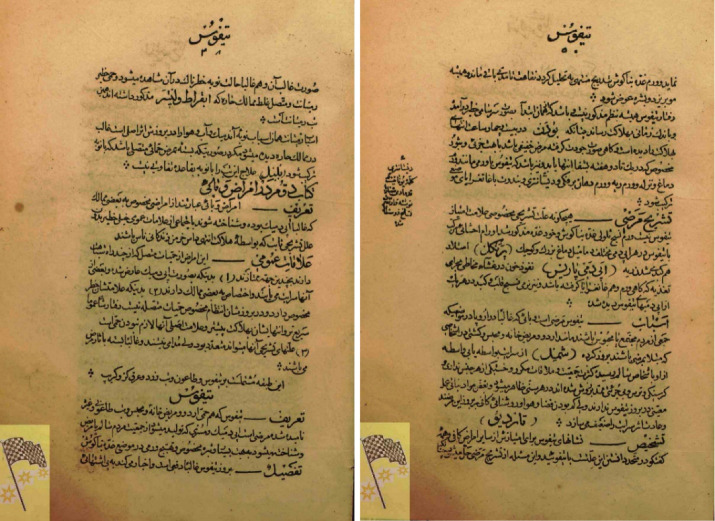
Matlaol-tebb Nasseri is a book in 581 pages in Persian written by Mirza Abolhassan Khan Ibn Abdul Wahab Tafreshi (1845–1902), It mentions the treatment of each disease and contains details about typhus.^7,34^

**Figure 3:**
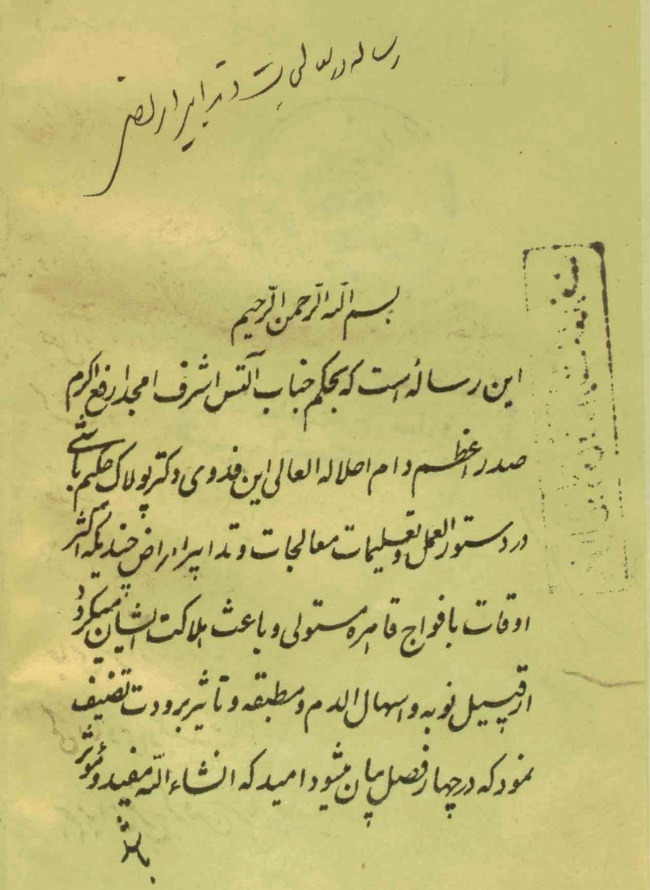
A 75-page treatise on the instructions for disease treatment and management was written by Dr. Jakob Eduard Polak in 1889 in Persian.^35^

 In 1918, Health Council officials suggested the word *Mohregheh* for typhus and *Motahbegheh* for typhoid fever to prevent misdiagnosis.^7^ In Iran, great efforts were made by the graduates and teachers of the Dar al-Fonun school, including the treatise of Tohfe-ye Naseri by Fakhr al-Atebba, Matlaol-tebb Nasseri by Mirza Abolhassan Khan Tafreshi and the treatise on instructions for disease treatment and management by Dr. Jakob Eduard Polak (Figures 1-3).

 Iranian Traditional medicine physicians also had their prescriptions and medications for typhus, typhoid, and similar diseases. According to Polak and Floor, Iranian physicians believed that prescribing venipuncture (phlebotomy) is considered to weaken and even accelerate death in *Mohregheh* (which was called typhus in the twentieth century) and used only enema and valerian for internal use,^6,8^ so they prescribed medication and nutrition instructions as:

 Medication instructions: Decoction of Violet, Jujube, Coriander seeds, green mung bean, and Mallow seeds as refined syrup.

 Nutrition instructions: Spinach and coriander soup or mallow leaves as strained.

 Care instructions: Protect the patient from cold, airflow, movement, and loud noises.^36^

 People initially considered it necessary to have some lice on their heads and bodies and considered it a sin to eradicate them, but over time they realized the role of lice in several diseases. To keep them away from themselves or their living place, they performed measures such as exposing their clothes and belongings to sunlight, boiling their clothes with tobacco dust, putting them on fire, washing their hair with tobacco water, and soaking their clothes in oil.^37^

 It was in 1909 that Howard Ricketts (1871–1910) discovered the underlying germ of the disease, and studied it with Stanislaus von Prowazekii (1875–1915). Both scientists contracted typhus and died.^7^

 Surprised by the discovery of the typhus-causing germ, Mostofi stated that “In the future, nothing will be discovered in medicine without any reason for the effect of so many germs in human health, because as we know, medicine is changing and has not yet proven anything. During the famine of 1917, eating raw vegetables was believed to be harmful to typhoid and typhus, and this carelessness was thought to transmit the disease, but today lice are considered to be carriers of the disease. So how do you know that camels will not be known to cause this disease tomorrow? Nature mysteries are too big and complex for man to claim he has discovered all of it so quickly and easily”.^18^

 It should also be known the first typhus vaccine was developed by the Polish zoologist Rudolf Weigl (1883 –1957) during the interbellum of the two world wars.^7^

## Conclusion

 Typhus was one of the most common diseases in Iran during the Qajar period. Of course, there were some therapeutic measures taken by European and Iranian physicians, but it seems that these measures were not enough. The peak of typhus in Iran was recorded in March 1918, in the midst of World War I, when it was killing 1,000 people per day in Tehran. The disease lasted until 1919 in Iran and took many lives. Typhus was usually overshadowed by two more deadly diseases, i.e., the Spanish flu and cholera, and did not receive much attention in those years. As a result, perhaps the typhus of 1918 can be considered a deadly disease erased from history. Although typhus broke out in Iran due to World War I and caused many deaths in Iran, the outbreak of typhus during World War II (1939–1945) is far better known than the typhus spread during 1917–1918. According to the narrations of oral history, this disease also spread during the ancient Iranian holidays (Nowruz), from March 21 to April 2, 1917.
