# Correction: Typhus Disease in Iran during the Qajar Period (1796 to 1925 AD); a Brief Historical Review

**DOI:** 10.34172/aim.34549

**Published:** 2025-05-01

**Authors:** Seyyed Alireza Golshani, Ghobad Mansourbakht

**Affiliations:** ^1^History of Iran after Islam, Department of History, Shahid Beheshti University, Tehran, Iran; ^2^Department of History, Shahid Beheshti University, Tehran, Iran

 This notice corrects the article titled “Typhus disease in Iran during the Qajar period (1725 to 1925 AD); a brief historical review. Arch Iran Med. 2022;25(11):758-764 (DOI: 10.34172/aim.2022.120).

 In the main article and the article titled, the address “Typhus disease in Iran during the Qajar period (1725 to 1925 AD); a brief historical review” has been updated to “Typhus disease in Iran during the Qajar period (1796 to 1925 AD); a brief historical review.” The Qājār dynasty ruled Iran from March 1796, with the formal coronation of Āghā Moḥammad Khān Qājār, until 31 October 1925. During this period, seven monarchs ascended the throne, making it a significant era spanning approximately 130 years in Iranian political history. The previous title mistakenly referred to this era as a 200-year span, an error that has now been corrected in light of accurate historical chronology, particularly the well-documented date of Āghā Moḥammad Khān’s enthronement.^1-4^

 Also funding segment in main document updated as:

 This work is based upon research funded by Iran National Science Foundation (INSF) under project No. 4020704 and on the date 2024-02-19. The Postdoctoral project to Under research support for conducting Department of History, Shahid Beheshti University, Iran. The present project work was conducted aiming to examine the role of shipping lines and international railways in accelerating the transmission of infectious diseases to the political borders of Qajar era Iran (1796 - 1925).

 These revisions have been implemented in both the PDF and HTML versions of the article.

## Correction Notice

 This notice addresses the article titled “Typhus Disease in Iran During the Qajar Period (1725 to 1925 AD); A Brief Historical Review” published in *Archives of Iranian Medicine* (2022; 25(11): 758-764, DOI: 10.34172/aim.2022.120).

 In the main article, the title has been updated from “Typhus Disease in Iran During the Qajar Period (1725 to 1925 AD); A Brief Historical Review” to “Typhus Disease in Iran During the Qajar Period (1796 to 1925 AD); A Brief Historical Review.” The Qājār dynasty ruled Iran from March 1796, with the formal coronation of Āghā Moḥammad Khān Qājār, until 31 October 1925. During this period, seven monarchs ascended the throne, making it a significant era spanning approximately 130 years in Iranian political history. The previous title mistakenly referred to this era as a 200-year span, an error that has now been corrected in light of accurate historical chronology, particularly the well-documented date of Āghā Moḥammad Khān’s enthronement.^1,2^

 Additionally, the funding segment in the main document has been updated to:

 “This work is based on research funded by the Iran National Science Foundation (INSF) under project No. 4020704, dated February 19, 2024. The postdoctoral project received scientific support from the Department of History, Faculty of Literature and Human Sciences at Shahid Beheshti University, Iran. This project aims to examine the role of shipping lines and international railways in accelerating the transmission of infectious diseases to the political borders of Qajar-era Iran (1796 - 1925).”

 These revisions have been implemented in both the PDF and HTML versions of the article.
